# The Impact of Clopidogrel Resistance on Clinical Outcome of Iranian Patients Undergoing Percutaneous Coronary Intervention

**Published:** 2018

**Authors:** Mohammad Haji Aghajani, Farzad Kobarfard, Seyed pouzhia Shojaei, Froozan Ahmadpour, Olia Safi, Neda Kazemina, Naeime Zarepishe, Mohammad Sistanizad

**Affiliations:** a *Department of Cardiology, Emam Hossein Teaching and Educational Center, Shahid Beheshti University of Medical Sciences, Tehran, Iran. *; b *Department of Medical Chemistry, Faculty of pharmacy, Shahid Beheshti University of Medical Sciences, Tehran, Iran. *; c *Department of Critical Care Medicine, Shahid Beheshti University of Medical Sciences, Tehran, Iran.*; d *Department of Clinical Pharmacy, Faculty of pharmacy, Shahid Beheshti University of Medical Sciences, Tehran, Iran.*

**Keywords:** Percutaneous coronary intervention, Drug resistance, Platelet aggregation, MACE, Light transmission aggregometer

## Abstract

The aim of current study was evaluating the frequency of clopidogrel resistance and its impact on clinical outcome of patients in Iranian patients. Patients undergoing percutaneous coronary intervention in Imam Hussein Medical center, Tehran, Iran, who received standard dosage of clopidogrel (Plavix®, Sanofi, France, 600 mg loading dose and 75 mg/day afterward) were recruited. Platelet aggregation was measured using light transmission aggregometer. The patients were categorized as responder (platelet aggregation less than 43%) and non-responder (platelet aggregation more than 43%). All patients were evaluated for major adverse cardio vascular events one month and 3 years after the angioplasty based on MACE criteria by phone contact. One hundred and five patients with average age of 60.30 ± 12.2 years entered the study of whom 26 (24.76%) did not respond to clopidogrel. None of patients experienced cardiac events one month after PCI. Three years after PCI, data were collected from 55 (69.62%) and 10 (38.46%) subjects in responder and non-responder groups, respectively. MACE criteria was positive in 4 patients, 3 (5.45%) in responder and 1 (10%) in non-responder group (*p* = 0.28). We did not find any significant differences between clopidogrel resistance and past medical history. In drug history 1 (1.26%) and 4 (15.38%) patients received omeprazole with clopidogrel in responder and none-responder group, respectively (*p* = 0.003). This study showed 24.76% resistance to clopidogrel in Iranian population but, we did not find any correlation between clopidogrel resistance and cardiac events in follow up maybe due to study limitations particularly missed follow-up in non-responder patients.

## Introduction

Cardiovascular diseases are the leading causes of death and disability in most countries of the world. Coronary heart disease (CHD) is the most common type of cardiovascular disease. (1) In the United States, CHD annually results in 502,000 deaths, and an economic burden of $133 billion. (2). Percutaneous coronary intervention (PCI) is a common non-surgical procedure used to open narrow or blocked coronary (heart) arteries and restore blood flow to the heart muscle. Major Adverse Cardiac Events (MACE) including stent-thrombosis, recurrent MI, death and need for urgent revascularization can occur during and after PCI. Activation and aggregation of platelets has an important role in the pathogenesis of these complications so, FDA currently recommends clopidogrel and aspirin for reduction of these events (3). 

Clopidogrel is a pro-drug that is predominantly catalyzed by cytochrome P450 (CYP) 2C19, 2C9, and 3A4 enzymes, (4, 5) and inhibits platelet activation through a selective and irreversible blockade of the adenosine diphosphate (ADP) P2Y12 receptors (6). Inter-individual variability in platelet response to clopidogrel, ranging from 5 to 44% (7-12) with clinical relevance (5, 8, 13-17) is well recognized. So, monitoring of platelet function to identify non-responders, which are in increased risk of MACE, could be useful. 

Adenosine diphosphate (ADP)-induced light transmittance aggregometry (LTA) is the gold standard test for evaluating platelet reactivity in patients receiving clopidogrel.(18) In this method platelet aggregation more than 43% induced by 5 mM ADP have been defined as clopidogrel non-responder (19). Data about clopidogrel resistance in Iranian population is scanty. The primary objective of this study was to investigate the prevalence of clopidogrel–resistance and secondary objective was evaluation of contributing risk factors to clopidogrel among Iranian patients. Also we evaluated patients MACE criteria 1 month and up to 3 years after PCI.

## Experimental

The population in this study was comprised of patients undergoing angioplasty with a drug eluting stent (DES) placement that were admitted to the coronary angiography, CCU, post-CCU and Cs-ICU of Imam Hossein Medical Center, affiliated to Shahid Beheshti Medical University, Tehran, Iran. All patients were given a written informed consent before entering the study, and the study was performed in accordance with the declaration of Helsinki and was approved by the Medical Ethics Review Board of Shahid Beheshti University of Medical Sciences. Patient were included in the study if they were subjected to angioplasty procedure with DES and consuming standard dosage of aspirin (75-325mg/day). The exclusion criteria were age under 18, history of clopidogrel usage in last 1 month, acute infarction in last 18 h platelet count < 100000/mm^3^, hematocrit < 25%, creatinine > 4mg/dL, usage of glycoprotein IIb/IIIa before procedure, history of alcohol consumption, previous PCI, contraindications for anticoagulant therapy, severe disease with life expectancy under 1 year, cancer and dialysis. 

Blood sampling from patients was performed from June 2011 to July 2013. For all patients entering the study, a questionnaire including demographic information including age, sex, past medical history (such as MI, HTN, HLP, DM, history of PCI, IHD), drug and habitual (such as smoking, addiction) history was completed and their correlation to clopidogrel resistance were evaluated with relative statistical tests. 

All patients received a loading dose of 600 mg clopidogrel (Plavix®, Sanofi, France) before the angioplasty and a daily dose of 75 mg afterward. Platelet aggregation test was performed for all patients 24 h after taking the loading dose of clopidogrel using light transmission aggregometry (LTA) method using a 4-channel Labtec APACT 4004 aggregometry and platelet aggregation equal and greater than 43% considered as clopidogrel non-responders.

*Statistical Analysis*

Data were analyzed by SSPS version 17.0 (SPSS, Inc., Chicago, IL, USA) with related tests. The results are expressed as a mean ± standard deviation (SD), and/or range. *p* values less than 0.05 were considered statistically significant. 

## Results

One hundred and five patients with mean age of 60.30 ± 12.2 entered the study. Sixty-four patients were female and the hypertension was medical history most common (66%) in subjects. Demographic findings, past medical and drug history of patients are summarized in Table 1. In our study, these factors including history of PCI, addiction, MI, IHD, HLP, smoking, DM, HTN do not have significant association with clopidogrel resistance.

**Figure 1:**
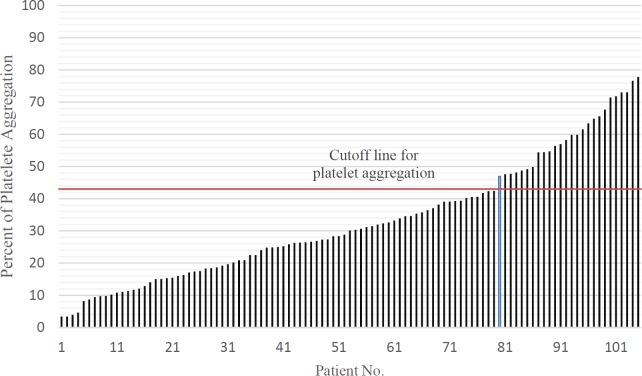
Distribution of platelet aggregation in study subjects 24 h after receiving clopidogrel.

The mean platelet aggregation in study population was 32.94% ± 18.83 (ranged from 3.41% to 77.79%). Twenty-six patients (24.76%) had platelet aggregation above 43% which were categorized as non-responder to clopidogrel. the data are shown in Figure 1.

**Table 1:** Demographic findings, past medical and drug history of 105 patients entering the study

Parameter	Total	Clopidogrel responder (No. = 79)	Clopidogrel non-responder (No.= 26)	*p*-value
**Age (mean ± SD)**	60.30±12.2	59.15 ± 11.4	63.8 ± 12.7	
**Female Sex; No. (%)**	64 (61%)	50 (63.29)	14(53.84)	0.23
**Smoker (No)**	24.76	29.11	11.54	0.07
**Addict (No)**	3.81	3.80	3.70	0.99
**PMH; No. (%)**				
**DM**	21(20.95)	16(20.25)	5(23.08)	0.75
**HTN**	69(65.71)	52(65.82)	17(65.38)	0.96
**IHD**	11(10.48)	9(11.4)	7.69	0.59
**HLP**	15(14.28)	11(13.92)	4(15.38)	0.85
**MI**	7(6.66)	5(6.32)	2(7.7)	0.8
**PCI**	5(4.76)	5(6.32)	0(0)	0.18
**Past Drug history**				
**PPIs**				
**Pantoprazole**	11(10.5)	11(13.92)	0(0)	0.04
**Omeprazole**	5(4.76)	1(1.26)	4(15.38)	0.003
**Atorvastatin**	64(60.95)	51(64.55)	13(50)	0.18
**Beta-blocker**	62(59.04)	48(60.79)	14(53.84)	0.53

One month and 3 years after PCI, patients MACE criteria were followed by telephone call. After one month, none of the patients experienced MACE in responder and non-responder groups. Three years after PCI, the data had been collected from 65 (61.90%) individuals, 55 (69.62%) of whom were in clopidogrel responder arm and 10 (38.46%) in non-responder group. MACE criteria was positive in 4 patients, 3 (5.45%) in responder and 1 (10%) in non-responder group. There was no significant difference between two arms in MACE criteria (*p* = 0.28). 

Also we analyzed the association of possible risk factors with resistance to clopidogrel. We did not find any significant differences between clopidogrel resistance and history of HTN, HLP, DM, PCI, IHD, and MI. 

In drug history 11 (13.92%) of patients in responder group were receiving pantoprazole while none of patients in non-responder group received this agent concurrent with loading dose of clopidogrel (*p* = 0.04). One (1.26%) and 4 (15.38%) patients received omeprazole with clopidogrel in responder and none-responder group, respectively (*p* = 0.003). There was no significant difference in other medications including atorvastatin, nitrate, and beta blockers in two groups.

## Discussion

The results of current study showed that the platelet function did not decreased to less than 43% in 24.76% of patients after receiving 600mg loading dose of clopidogrel. We did not detect any significant difference in MACE criteria in responder and non-responder patients in phone call follow-up 1 month and 3 years after PCI. Furthermore, we did not find any correlation between patient’s characteristics and response to clopidogrel, except treatment with omeprazole. 

In a systematic review by Snoep *et al*. mean unadjusted prevalence of clopidogrel resistance, weighted for study size, reported to be 21 % (95% CI, 17%-25%) which is in accordance with results of our study(20). Lapantalo *et al*. evaluated 1608 Finnish patients and reported 20% resistance among which is line of with our study (21). In German population, Schulz *et al.* and Geisler el al. reported 20% and 5.8% resistance to clopidogrel respectively. (22-24). Although, the results of study by Geisler el al. differ from our reported percent, it is consistent with those of Schulz *et al.*

In Iranian population in 2013, our team measured platelet aggregation pre and post treatment with clopidogrel in 31 patients and divided patients into three groups. The results showed that 22% and 13% of the patients were semi-resistant and none-responder to clopidogrel, respectively (12). Also our results are in agreement with Namazi’s (2012) findings which showed 26% of clopidogrel non-responsiveness, in Iranian population (25).

In contrast to earlier findings, however, no evidence of relationship between underlying disease including DM, HTN, IHD, PCI, MI, and habitual history including addiction and smoking was detected in our study. Al-Azzam *et al*. evaluated the factors that contribute to clopidogrel resistance and reported that use of calcium channel blockers and low HDL have significant association with clopidogrel resistance while age, diabetes, hypertension, smoking and aspirin use did not have significant contribution to clopidogrel resistance (24). In another study, ZHANG chun-ying *et al*. observed significant association between clopidogrel resistance and history of smoking, diabetes, fasting plasma glucose, glycosylated hemoglobin, triglyceride, and cardiac troponin (26). 

 de Miguel Castro, A., *et al*. (2009) showed that lower response to clopidogrel were significantly associated with MACE occurrence in 1-year follow-up (27). This differs from the findings presented here maybe due to study limitations particularly missed follow-up in non-responder patients which consisted of more than 60% of the subjects. The other limitation of current study is its single center design and small sample size for detection of MACE criteria. 

## Conclusion

The results of this investigation show that there is no major difference between Iranian population and other studies regarding the resistance to clopidogrel. The other findings were lack of correlation between clopidogrel resistance and past medical and drug history. Also the MACE criteria was not different between responders and non-responders in the current study. Due to the study limitations, further multi center investigations with higher number of patients and accurate follow up of the patients is recommended.
